# From Dukes-MAC Staging System to Molecular Classification: Evolving Concepts in Colorectal Cancer

**DOI:** 10.3390/ijms23169455

**Published:** 2022-08-21

**Authors:** Laura Banias, Ioan Jung, Rebeca Chiciudean, Simona Gurzu

**Affiliations:** 1 Department of Pathology, George Emil Palade University of Medicine, Pharmacy, Science and Technology, 38 Gheorghe Marinescu Street, 540139 Targu Mures, Romania; 2 Research Center of Oncopathology and Transdisciplinary Research (CCOMT), George Emil Palade University of Medicine, Pharmacy, Science and Technology, 540136 Targu Mures, Romania

**Keywords:** Dukes MAC staging, colorectal cancer, molecular classification, TNM, epithelial–mesenchymal transition

## Abstract

This historical review aimed to summarize the main changes that colorectal carcinoma (CRC) staging systems suffered over time, starting from the creation of the classical Duke’s classification, modified Astler–Coller staging, internationally used TNM (T—primary tumor, N—regional lymph nodes’ status, M—distant metastases) staging system, and ending with molecular classifications and epithelial–mesenchymal transition (EMT) concept. Besides currently used staging parameters, this paper briefly presents the author’s contribution in creating an immunohistochemical (IHC)-based molecular classification of CRC. It refers to the identification of three molecular groups of CRCs (epithelial, mesenchymal and hybrid) based on the IHC markers E-cadherin, β-catenin, maspin, and vimentin. Maspin is a novel IHC antibody helpful for tumor budding assessment, which role depends on its subcellular localization (cytoplasm vs. nuclei). The long road of updating the staging criteria for CRC has not come to an end. The newest prognostic biomarkers, aimed to be included in the molecular classifications, exert predictive roles, and become more and more important for targeted therapy decisions.

## 1. Introduction

Colorectal cancer, being mostly colorectal carcinomas (CRC), represents the third most diagnosed cancer and the second cause of cancer-related death [1]. Over the years, numerous studies focused on various aspects regarding risk factors, carcinogenesis, diagnostic markers, procedures, staging, and therapy. Although some questions have been answered and important mechanisms have been deciphered, further research is constantly needed, aiming to discover new prognostic markers, diagnostic methods, therapeutic agents, and an updated, more optimized staging system [2,3,4].

The current review is focused on the CRC staging systems, from the classical Dukes’ classification to the TNM (T—primary tumor, N—regional lymph nodes, M—distant metastases) stages included in the latest published edition of the American Joint Committee of Cancer (AJCC), with continuous updates over the past decades, together with relevant prognostic markers used by recent studies in the search for molecular classification of CRC, including our team’s contribution to the field.

## 2. Methodology

For this review of internationally used staging systems, peer-reviewed publications identified on PubMed, Scopus and web of science databases using as keywords colorectal carcinoma AND “staging”, “staging system Dukes”, “staging Astler Coller”, “staging system AJCC”, and “TNM” or “molecular classification” were included. The databases were searched from inception to 4 July 2022.

Only articles written in the English language, based on human tissue studies, with available abstract and full text, were taken into consideration. Staging-related data were also extracted from older versions of the AJCC manuals available online at https://cancerstaging.org/references-tools/deskreferences/pages/default.aspx (accessed on 3 July 2022). After the title’s evaluation, the selection required checking the abstracts and, in the end, the full-text variant of the articles was read. 

After deduplication, the initial search resulted in 659 papers. After removing letters to the editor, and articles with unavailable full text in English, a number of 286 papers was selected for full-text screening and 66 of them were included in the present review.

## 3. Dukes-MAC Era

Staging systems are used to enable the prediction of survival, an internationally appropriate and uniform case evaluation, and treatment decision. Dukes proposed in 1932 the first staging system of CRC, starting with the rectum and then for colorectal segments [5,6]. The first variant of Dukes’ classification included three stages, based on the extent of tumor spread. Stage A represented tumors limited to the rectal wall, stage B dedicated to those that go beyond the wall, but without lymph node (LN) metastases and C for those with positive LN [5,6]. Three years later, stage C was divided into C1 and C2, depending on the location of metastatic LN—regional ones (C1) or LNs located beyond the level of hemorrhoidal/inferior mesenteric vessels ligature (Table 1) [6,7,8]. 

Further subclassification and modification of these stages were conducted by Astler and Coller (MAC) in 1954. They proposed splitting former stage A into A and B1. Only superficial tumors limited to the mucosa were included in stage A and those infiltrating the submucosa, but not crossing muscularis propria in stage B1. Ex-stage B becomes B2. No metastases were included in stages A, B1 and B2. Cases with positive LNs were included in C stages, respectively, C1 and C2, corresponding to B1 and B2 with associated LN metastases (Table 1) [7,8,9].

Turnbull proposed in 1967 a new stage—stage D—for tumors with distant systemic spread or direct invasion of the peritoneum—which is partially equivalent to the TNM stage IV introduced in 1977 (Table 1 and Table 2) and kept in the AJCC manual [7,8,10,11]. Crossing of the colorectal wall or direct invasion of the surrounding structures is considered a distinct stage from 1974 when Gunderson and Sosin proposed stage B3 for cases without LN metastases and C3 for those with positive LNs (Table 1 and Table 2) [7,8,9,12].

Although new parameters and sub-divisions were further included in the TNM staging system, the Dukes paradigm of considering lymph nodes as one of the strongest prognostic parameter is kept even in the 8th edition of the AJCC Manual [13].

## 4. TNM-Based Staging System

From 1977 until nowadays, the well-known and internationally utilized pathological TNM (pTNM) staging suffered periodical changes which are included in well-known AJCC manuals (Table 2). Continuous stage refinement represents a necessity due to differences in patient survival correlated with various parameters, proved by multiple studies [7,11,14,15]. An evidence-based medicine group was created in 2013 to establish the potentially new content’s level of evidence so that only level I-III data were included in the last edition of AJCC [11,16].

The most significant changes were added starting with the 6th edition of AJCC in 2002. Although the T, N, and M parameters were not modified, following the Dukes paradigm, cases were sub-classified based on the number of metastatic nodes and the clinical stages I, II and III was suggested to be included in the histopathological reports [13]. For therapeutic purposes, the guidelines of the European Society of Medical Oncology (ESMO) grouped patients from stage III into low (T1-3N1) and high-risk (T4 or N2). This sub-grouping serves for the length of chemotherapy (short or prolonged) and prognostic assessment [17].

Problems regarding the influence of pre-operative therapy were also firstly addressed in the 6th edition of AJCC with the addition of the letter “y” in front of the pTNM stage, with no other evaluation differences compared to untreated tumors [14]. Precise criteria for appreciation of response to chemoradiotherapy were included and perfected in the next two staging manuals, represented by Ryan’s scheme which grades tumor regression as grade 0 (complete response, with no identifiable tumor cells), 1 (a nearly complete response to therapy with evident tumor regression displaying only single tumor cells or rare small groups of tumor cells), 2 (partial response, when more than single tumor cells/small groups of tumor cells are still present, but regression is noticeable) or 3 (poor response or no response, with no tumor regression and presence of tumor cells in over 50% of the examined tissue) [11,15,18,19].

Another useful aspect refers to the presence of multiple synchronous colorectal tumors. It is represented by the symbol “(m)” inserted at the end of the pTNM stage [14].

In the last two editions of the AJCC manuals, it was included in the stage N1c for cases with the presence of tumor deposits in the absence of LN metastases (7th edition), based on a more unfavorable prognosis compared with N0 staged cases. A distinct M1c stage was proposed for the presence of peritoneal carcinomatosis (8th edition). It was based on the worse outcome of these cases compared with those spreading in other organs. The T4 stage was also sub-divided into T4a and T4b (Table 3 and Table 4) [11,16,20,21,22].

Currently, it is indicated to include in the histopathological reports, along with the TNM stage criteria (Table 2), those prognostic parameters which can be identified after macro- or microscopic assessment. It is about the presence/absence of lymphovascular and/or perineural invasion of the tumor cells, high-grade tumor, the status of the resection margins (R0—tumor-free margins, R1—microscopically identified tumor invasion of the margins, R2—macroscopic evidence of margin infiltration), perforation, obstruction, number of examined lymph nodes and preoperative serum level of carcinoembryonic antigen (CEA). If few than 12 lymph nodes were harvested the risk of recurrence is higher, especially for poorly (G3) or undifferentiated tumors which are considered high-grade carcinomas [23].

As the systemic inflammatory response (SIR) plays role in carcinogenesis, more and more studies are focused on the prognostic role of the SIR-related parameters such as neutrophil-to-lymphocytes ratio (NLR) or lymphocytes-to-monocytes ratio (LMR). LMR represents the ratio between preoperative lymphocyte and monocyte counts assessed at baseline. LMR and NLR values are correlated with the TNM stage. High preo-operative NLR (over 3.11) and low LMR are indicators of poorer overall survival rates [24,25].

## 5. Macroscopic Assessment-Mesorectal Fascia

Rectal cancer accounts for one-third of all CRCs. There are some aspects that regard this segment only. Starting with the last (8th) edition of AJCC, it is recommended, for rectal carcinomas, to evaluate the quality of mesorectal fascia [7,11,14]. The peritumoral mesorectum can either be complete, nearly complete, or incomplete, based on the outer surface’s aspect (smooth, irregular, or in small quantity), presence of defects in the mesorectal adipose tissue (less than 5 mm, more than 5 mm but without exposing the outer muscle layer of the rectal wall or with visible muscularis propria), grade of coning (none, moderate, marked) and the aspect of the circumferential mesorectal resection margin (regular or irregular) [11,26]. There is a direct association between the quality of mesorectal excision and the status of circumferential margin (either open surgery or non-invasive procedures), respectively, the risk of tumor recurrence. Complete removal with intact fascia (R0 resection), which is also known as total mesorectal resection (TME) represents an independent favorable prognostic factor directly correlated with a recurrence-free survival rate [11,26,27,28]. As high NLR and low LMR were correlated with a high SRI and incomplete fascia, these pre-operatively serum indicators can guide surgeons to choose the best therapeutic approach [24].

## 6. Preoperative Imagistic Assessment-Particular Issues

Preoperative imagistic evaluation of CRC can be performed with computed tomography (CT), magnetic resonance imaging (MRI) and positron emission tomography (PET) combined with CT (PET/CT). They have advantages and limitations. CT is useful for lymph node assessment. MRI is mainly used for checking a suspicion of relapses, especially for rectal tumors and suspected hepatic metastases. PET/CT is indicated to evaluate the whole body and check distant metastases [29].

**Figure 1:**
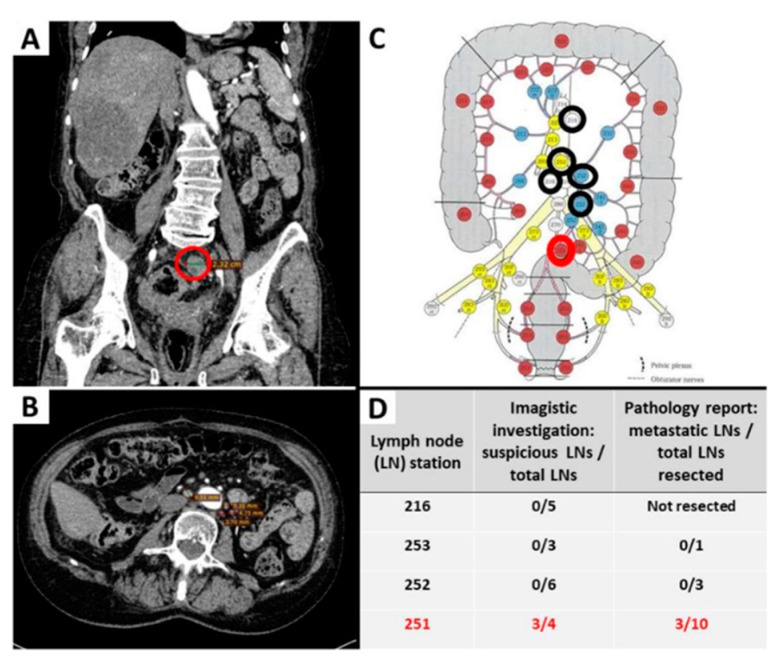
Evaluation of the lymph node status before and after surgical intervention for removal of a rectal carcinoma. Examples of imagistically identified LNs are shown on abdominopelvic CT scan, coronal (**A**) and axial view (**B**). Suspect LNs were present only in the perirectal group 251, the largest one measuring 23 mm ((**A**,**C**), highlighted with a red circle), confirmed as metastatic LNs on histopathological examination (table (**D**)). Other identified LNs were homogenous, measuring less than 10 mm, considered non-suspicious ((**B**)—example of periaortic LNs), marked with a black circle on the map (**C**), and correlated with the absence of metastasis after microscopic evaluation (**D**). The map with lymph node stations was adapted by our team with permission from Yamamoto S et al. [30,31].

Proper staging of CRC requires a precise evaluation of LN status. It should be performed by a transdisciplinary team and start before surgery with CT or MRI scans. The suspect LNs are evaluated based on imagistic criteria such as size (less than 5 mm, between 5 and 10 mm or greater than 10 mm), shape, contour, and heterogeneity [30]. Afterward, the LN stations map that was published in the 3rd edition of the Japanese Classification of Colorectal, Appendiceal, and Anal Carcinoma can be used by the radiologist to encircle the imagistically identified LN groups (Figure 1). This adapted map is meant to assure the surgical removal of all suspect LNs, thus avoiding pathological sub-staging [29,30]. Based on such maps, new valid evidence-based results will help future staging updates [31,32,33]. CT provides a lower performance, compared with MRI, for evaluation of the depth of infiltration, especially for low-T stages [29].

For rectal cancers, pelvic MRI is the gold standard imaging way for evaluation of primary tumors and local recurrences, even for cases with a large post-radiotherapy fibrotic scar. The risk of local relapse can be predicted based on the distance between the tumor and circumferential resection margins, combined with the presence or absence of extramural invasion [29].

Further changes and ways of perfecting the evaluation methods as a response to current challenges in the diagnostic and therapeutical case management are continuously being studied and new valid evidence-based results will help future staging updates [32,33,34].

For a proper MRI evaluation of the TME, the newest classification beyond TME (BTME) was recently proposed. After pelvic MRI, cases can be grouped based on their localization in the eight compartments: 1. Anterior above peritoneal reflection (sigmoid colon, small bowel, ureters, iliac vessels above peritoneal reflection, lateral pelvic sidewall fascia); 2. Anterior below Peritoneal Reflection (Genitourinary organs and pubic symphysis); 3. Central (Rectum and perirectal fat); 4. Posterior (Coccyx, pre-/retro-sacral area, sciatic nerve); 5. Lateral (Internal and external iliac vessels, lateral pelvic lymph nodes, piriformis and internal obturator muscles); 6. Infralevator (levator ani muscles, external sphincter, ischio-anal fossa); 7. Anterior urogenital (Perineal, vaginal, distal urethra, crus penis). The worse survival was reported for patients with tumors located in the first compartment (anterior below peritoneal reflection) same as for those with tumors involving multiple compartments [35].

[18F]-FDG PET combined with MR ([18F]-FDG PET/MR) was recently proved to have high specificity and sensitivity for the diagnosis of CRC, evaluation of the free margins (distance from tumors) and identification of distant metastases. Due to limited spatial resolution, the preoperative T stage cannot be properly performed with PET/CT. High specificity, but low sensitivity was also proved for N staging [29].

## 7. Molecular Classification

### 7.1. Consensus Molecular Subtype Classification

Differences in tumor behavior and response to therapy in same-stage CRC cases have increased the need for gene-expression studies and the creation of a molecular classification that would facilitate targeted therapy [2,4,36,37,38,39]. In this regard, four consensus molecular subtypes (CMS 1-4) were introduced in 2005, based on multiple molecular characteristics and the presence or absence of epithelial-mesenchymal transition (EMT) [39].

Tumors belonging to the CMS1 subtype are hypermutated, with BRAF mutant status, microsatellite instability (MSI-H), and an important immune reaction. CMS1 group is also known as MSI immune. Carcinogenesis seems to be driven via JAK-STAT and PD-1 signaling pathways [39,40]. Although the pathways are similar for MSI and MSS cases belonging to this group, MSS carcinomas’ behavior and answer to therapy are also influenced by CD8+ cytotoxic T cell infiltration amount [40].

CMS2 (canonical) and CMS3 (metabolic) represent epithelial subtypes. CMS2 is chromosomally unstable, with activation of WNT and MYC signaling pathways. CMS3 shows metabolic deregulations and KRAS mutations and comprises MSI-H and one-third of cases that are microsatellite stable (MSS). The CMS3 MSS-carcinomas are architecturally such as MSI tumors [36,37,38,39,40,41]. 

CRCs with stromal invasion, angiogenesis, and transforming growth factor β (TGF-ß) activation are included in the CMS4 subtype, which is also known as the mesenchymal subtype [36,37,38,39,40,41]. Hypermethylation of the miR-200 family’s promoter was associated with stimulation of the EMT process in this mesenchymal subtype, frequently diagnosed in advanced stages and associated with worse survival parameters and activation of vascular endothelial growth factor (VEGF) and TGF-ß [38,42]. A risk stratification formula based on the expression of six immune genes, recently described by Zhang et al. might become useful in the clinical management of CMS4-type CRC [43].

In one of the recent studies, a refined classification of the CMS2 and CMS3 (epithelial cases) was proposed based on intrinsic epithelial subtype (I), microsatellite instability status (M) and fibrosis (F). It was called “iCMS” or “IMF” classification but implementation in daily practice is not easy to be performed [41].

Studies confirm response and outcome differences between tumors included in the four CMSs, larger cohorts being required for any valid official changes [44,45,46,47]. CMS1, MSI immune subtype, mostly identified in CRC of the right colon, seems to respond well to immunotherapy and to show better prognosis when bevacizumab, a VEGF inhibitor, is associated with the classical treatment scheme [37,44]. Although immunotherapy shows promising results for MSI-H cases from the CMS1 group, the CMS1-MSS carcinomas do not respond to immune checkpoint inhibitors [40].

Heterogenous research results indicate better overall survival (OS) for CRCs CMS2 and CMS3 when bevacizumab or cetuximab, an epidermal growth factor receptor (EGFR) inhibitor, is associated with classical therapy [37,44,45]. The latter also showed significant benefits when used for BRAF/RAS wild type, left-sided metastatic CRCs [42]. Adding cetuximab or irinotecan to CMS4-CRC chemotherapy appears to be more beneficial than adding bevacizumab or oxaliplatin-based therapy [47,48]. As KRAS mutations can be identified in CMS4 carcinomas, resistance to cetuximab should be considered [40].

Besides aiding the molecular classification process, microsatellite status by itself shows important diagnostic and therapeutic implications. It represents the presence of repeated sequences encompassing 1-6 nucleotides, causing mutations of the DNA mismatch repair (MMR) genes (MLH1, MSH2, MSH6, and PMS2), mutations that can be inherited (Lynch syndrome) or developed sporadically [36,37]. Screening for mutations of these genes or loss of IHC expression of their corresponding proteins enables the selection of MMR-deficient/MSI-high tumors, which are known to respond to fluoropyrimidine-based therapy and immunotherapy (pembrolizumab and nivolumab being recently approved by the Food and Drug Administration) [36,37,38,41].

### 7.2. Immunohistochemical-Based Molecular Classification

Multiple studies attempted to molecularly classify CRC using the expression of IHC antibodies for legit reasons such as cost-efficiency and availability in most pathology departments [48,49,50,51,52]. Most research studies used the following panel of antibodies: cytokeratin, CDX2 for epithelial-like tumors, FRMD6, ZEB1, HTR2B for mesenchymal-like tumors, and determination of microsatellite status [50,52,53].

These stains were not enough for the distinction between CMS2 and CMS3, which are mainly driven via the Wnt pathway [40]. Li X. et al. recently added β-catenin to the above-mentioned panel, considering positive nuclear expression an indicator of CMS2, because CTNNB1, the gene encoding β-catenin, appeared to be upregulated in this subtype [49].

**Figure 2:**
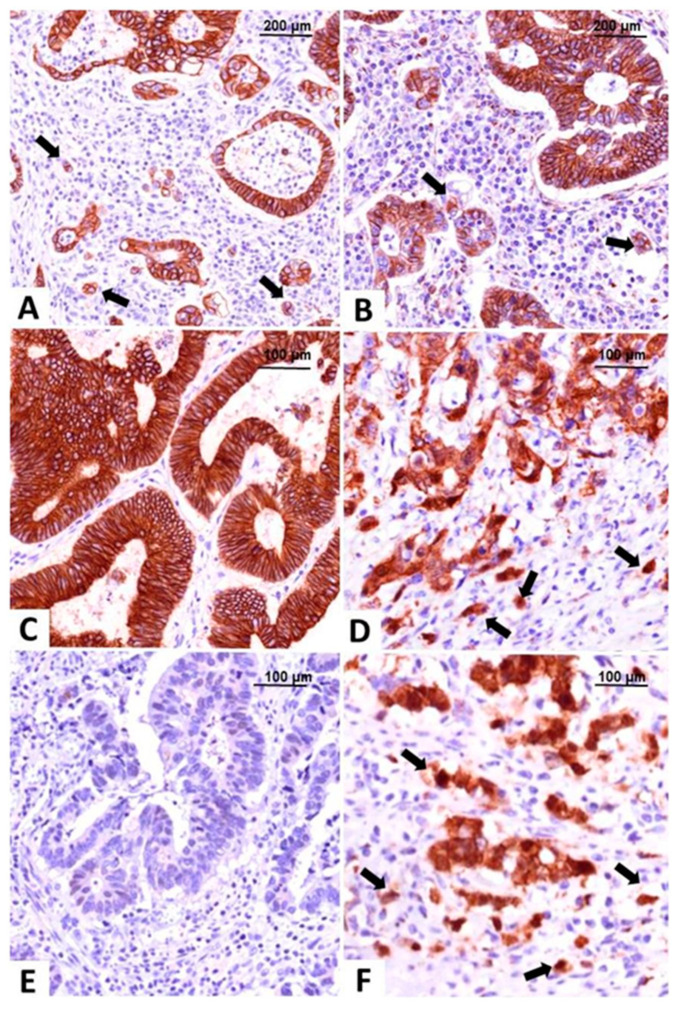
Molecular classification of colorectal carcinomas based on the immunohistochemical expression of E-cadherin and β-catenin. The epithelial subtype is easily recognized by diffuse membrane staining for E-cadherin (**A**) and β-catenin (**B**), in the core and tumor buds (indicated with arrows). The intermediate, hybrid subtype, presents epithelial-type expression in the tumor center, with membrane expression of E-cadherin (**C**) and β-catenin (**D**), and buds with mesenchymal immunophenotype showing nuclear β-catenin, indicated with arrows (**D**). The mesenchymal subtype does not stain for E-cadherin (**E**) and β-catenin (**F**) is predominantly nuclear, in both tumor center and buds, indicated with arrows (**F**). Pictures from the personal collection of authors—referenced data published in 2020–2021 [51,56].

Our team also focused on classifying CRC based on IHC reactions and used markers of EMT such as E-cadherin, β- catenin, vimentin, and maspin, evaluated in both tumor center and invasion front/tumor buds (Figure 2) [51,54,55]. We contoured three subtypes: epithelial (diffuse membrane expression of E-cadherin and β-catenin associated with negative vimentin), mesenchymal (loss of E-cadherin expression, positive vimentin and nuclear staining of β-catenin and maspin) and one with mixed epithelial-mesenchymal features called hybrid (epithelial-like pattern in the tumor center and mesenchymal characteristics in the invasion front), all of them exemplified in Figure 2 [51,56].

Tumor budding, defined as the single tumor cells or groups of no more than four tumor cells identified in the invasion front, represents an extensively studied parameter with a Hematoxylin-Eosin +/− cytokeratin slide-based evaluation protocol published in 2017, represents an independent prognostic marker not yet included in AJCC staging manual, but its importance and suggestion for addition in the pathological report are mentioned in oncological practice guidelines for both colon and rectal carcinomas [55,56,57,58,59,60]. For a better assessment of budding degree, our team used maspin’s expression which is in the nucleus at the level of the tumor buds and helps with their identification even on the background of an abundant inflammatory stroma [56,61,62,63].

Evaluation of subcellular maspin’s expression, combined with microsatellite status, could also be of therapeutic relevance [56,64]. Cytoplasmic staining identified in serrated MSI carcinomas might indicate favorable prognosis, while nuclear expression evaluated in microsatellite stable carcinomas is associated with high-grade tumor budding, EMT, mesenchymal subtype, worse prognosis, and could indicate response to therapy with fluorouracil [54,63,64]. 

Proved to be related to EMT and tumor-associated angiogenesis, maspin is opening a window for potential targeted therapy [56,62,65,66,67].

### 7.3. Precision Medicine

Like other tumors, it is thought that, in the near future, the therapy of CRC will be completely based on molecular diagnostic tests. Deep learning machines can already be used for the evaluation of whole slide images and establishing histological grade, budding degree or other prognostic parameters [18].

The role of pathologist needs to be revisited and next-generation sequencing platforms will replace large parts of ancillary tests. However, as most of the molecular tests are performed from paraffin-embedded tissues, the tissue quality still depends on the pre-analytical processing. Identification of the tumor-rich areas also depends on the pathologist and its role remains crucial for proper staging and lymph node harvesting [67].

## 8. Summary and Future Perspectives

This review shorty presented the historical evolution of CRC staging systems, using detailed tables to highlight the main modifications and the current interest in molecular classification. It aims for better stratification of cases, above classical staging limitations, in constant search for prognostic biomarkers with beneficial therapeutic impact. Promising discoveries have been made, but further studies are necessary to validate these achievements and include them in future staging manuals.

## Figures and Tables

**Table 1:** Localized colorectal carcinomas: Dukes MAC versus AJCC staging system.

Dukes	MAC (Modified Astler–Coller)	Stages According to the 8th Edition of AJCC	
-	-	0 (Tis, N0)	M0
A = tumor confined to the rectal wall (1932)	A = limited to the mucosa	I (T1-2, N0)	
	B1 = infiltration of the submucosa, but not through muscularis propria, without LN metastases	II A (T3, N0)	
B = tumor infiltrates extra-rectal tissues (1932)	B2 = crossing muscularis propria, without LN metastases	II B (T4a, N0)	M0
	B3 = lesions invading through the colorectal wall, adhered to/invading adjacent structures/organs, without LN metastases (Gunderson and Sosin, 1974)	II C (T4b, N0)	

Based on references [5,6,7,8,9,10,11,12].

**Table 2:** Metastatic colorectal carcinomas: Dukes MAC versus AJCC staging system.

Dukes	MAC (Modified Astler–Coller)	Stages According to the 8th Edition of AJCC		
C = tumor with regional lymph nodes metastases (1932)	C1 = B1 + LN metastases	IIIA	T1-2, N1/N1c	M0
			T1, N2a	
C1 = metastasis in lymph nodes (LN) close to the primary tumor (1935)	C2 = B2 + LN metastases	IIIB	T3-4a, N1/N1c	
	C1		T2-3, N2a	
	C2		T1-2, N2b	
C2 = involvement of the LN stations up to the main ligature of the superior hemorrhoidal/inferior mesenteric vessels (1935)	C2	IIIC	T4a, N2a	
	C2		T3-4a, N2b	
	C3 = B3 + LN metastases (Gunderson and Sosin, 1974)		T4b, N1-2	
-	D = distant spread/peritoneal invasion/infiltration of adjacent organs (Turnbull, 1967)	IV A (any T, any N, M1a)		
		IV B (any T, any N, M1b)		
		IV C (any T, any N, M1c)		

Based on references [5,6,7,8,9,10,11,12].

**Table 3:** Changes of colorectal carcinomas staging system (pT = depth of tumor infiltration) according to AJCC (based on references [7,8,11,13,14,15] and AJCC manuals accessed at https://cancerstaging.org/references-tools/deskreferences/pages/default.aspx (accessed on 3 July 2022)).

AJCC Editions (Year of Publication)						
	1st (1977)	2nd (1983)	3rd (1988)	4th (1992), 5th (1997), 6th (2002)	7th (2009)	8th (2016)
**T—primary tumor**	**Tx** = the tumor cannot be evaluated					
	**T0** = no evidence of primary tumor					
	**Tis** = no invasion in lamina propria			**Tis** = intraepithelial tumor or invasion of the lamina propria, no extension through the muscularis mucosae		
	**T1** = confined to the mucosa or submucosa		**T1** = tumor invades submucosa			
	**T2** = infiltration of the muscle wall or serosa, but no extension beyond	**T2a** = invasion of muscularis propria	**T2** = tumor invades muscularis propria			
		**T2b** = complete penetration of the muscularis propria				
	**T3** = invasion of all the layers, with extension to adjacent structures or organs, but without fistula	**T3** = invasion of all the walls layers, including serosa, +/− extension in the nearby organs, +/− fistula	**T3** = invasion of the subserosa or the nonperitonealized pericolic or perirectal tissues			
	**T4** = presence of a fistula	**T4** = direct extension beyond contiguous tissue or the adjacent organs	**T4** = invasion of the visceral peritoneum and/or direct spread to other organs or structures (including other colorectal segments)		**T4a** = invasion of the visceral peritoneum	**T4a** = invasion of the visceral peritoneum, including cases with perforation
	**T5** = tumor which has spread beyond the adjacent organs				**T4b** = direct invasion/adherence to other organs or structures	

**Table 4:** Changes of colorectal carcinomas staging system (metastatic stations) according to AJCC (based on references [7,8,11,13,14,15] and AJCC manuals accessed at https://cancerstaging.org/references-tools/deskreferences/pages/default.aspx (accessed on 3 July 2022)).

AJCC Editions (Year of Publication)						
	1st (1977)	2nd (1983)	3rd (1988)	4th (1992), 5th (1997), 6th (2002)	7th (2009)	8th (2016)
**N—regional lymph nodes** **(LN)**	**Nx** = regional lymph nodes cannot be assessed					
	**N0** = no metastasis in the regional LN					
	**N1** = regional LN involved, those distal to inferior mesenteric artery	**N1** = 1-3 LN adjacent to the primary tumor	**N1** = 1–3 pericolic or perirectal LN		**N1a** = one regional LN	
					**N1b** = 2–3 regional LN	
					**N1c** = no LN metastases, but tumor deposits identified	
		**N2** = LN involved extending to the line of resection or ligature of blood vessels	**N2** = 4 or more pericolic or perirectal LN		**N2a** = 4–6 regional LN	
					**N2b** = 7 or more regional LN	
		**N3** = LN with metastasis, but location not identified	**N3** = metastasis in any LN along the course of a vascular trunk	**N3** = in any LN along the course of a vascular trunk or apical node (if marked by the surgeon)—removed in 5th edition		
**M—distant metastasis**	**Mx** = not assessed					
	**M0** = no distant metastasis					
	**M1** = distant metastasis (including other LN than the regional ones and carcinomatosis)				**M1a** = in one organ/site	**M1a** = in one organ/site, without peritoneal metastases
					**M1b** = multiple distant metastatic sites or to the peritoneum	**M1b** = in 2 or more distant sites/organs, without peritoneal metastases
						**M1c** = peritoneal carcinomatosis
